# From Genes to Transcripts, a Tightly Regulated Journey in *Plasmodium*

**DOI:** 10.3389/fcimb.2020.618454

**Published:** 2020-12-17

**Authors:** Thomas Hollin, Karine G. Le Roch

**Affiliations:** Department of Molecular, Cell and Systems Biology, University of California Riverside, CA, United States

**Keywords:** *Plasmodium*, gene regulation, epigenetics, chromatin, AP2-G, sexual commitment

## Abstract

Over the past decade, we have witnessed significant progresses in understanding gene regulation in Apicomplexa including the human malaria parasite, *Plasmodium falciparum*. This parasite possesses the ability to convert in multiple stages in various hosts, cell types, and environments. Recent findings indicate that *P. falciparum* is talented at using efficient and complementary molecular mechanisms to ensure a tight control of gene expression at each stage of its life cycle. Here, we review the current understanding on the contribution of the epigenome, atypical transcription factors, and chromatin organization to regulate stage conversion in *P. falciparum*. The adjustment of these regulatory mechanisms occurring during the progression of the life cycle will be extensively discussed.

## Introduction

Malaria affected 228 million people and 405,000 deaths in 2018 (WHO, 2019) and remains one of the major global health problems. In sub-Saharan Africa, this disease is responsible for ~20% of all-cause mortality among children under 5 years old. The causative agent is a protozoan parasite, *Plasmodium*, belonging to the phylum Apicomplexa. Although, five plasmodial species can infect human, *P. falciparum* is associated with the greatest morbidity and mortality.

The life cycle of *P. falciparum* is complex and can be divided in two parts: the sexual phase in mosquito vector and asexual phase in human liver and red blood cells. After a bite by an infected female Anopheles, sporozoites are injected into the human and invade hepatocytes. This asymptomatic phase leads to the multiplication of parasites, which are released into the bloodstream and initiate the intraerythrocytic developmental cycle (IDC) inside red blood cells. During this cycle, merozoite develop to immature ring and progress to mature schizont, before dividing into 16 to 32 new merozoites, which immediately invade new erythrocytes. A portion of these parasites differentiate and mature into male and female gametocytes, and are ingested by another female mosquito. Thousands of sporozoites will be produce and migrate to the salivary glands to complete the cycle.

This parasite life cycle progression is accompanied by important transcriptional changes at the population level (Bozdech et al., 2003; Le Roch et al., 2003; Lemieux et al., 2009; Otto et al., 2010; López-Barragán et al., 2011; Hoo et al., 2016; Toenhake et al., 2018). Recently, advances in single-cell RNA sequencing provided new insights in our understanding of gene expression across different stages and *Plasmodium* species (Reid et al., 2018; Walzer et al., 2018; Howick et al., 2019; Sà et al., 2020). To ensure this dynamic gene expression, adjustable according to environmental factors and the parasite development, a tight and coordinated regulation is fundamental at each stage. Despite recent progress, a complete understanding of all mechanisms regulating gene expression is still lacking and several biological questions remained to be answer if we want to find new way to hamper parasite proliferation in a specific manner. Here, we discuss the current knowledge on the involvement of regulatory mechanisms in gene expression across the parasite development and stage conversion. The contribution of epigenetics and chromatin-associated proteins as well as the role of chromatin organization will be detailed, with a particular interest in *P. falciparum*.

## Overview of Chromatin Structure and Gene Regulation in Plasmodium

In this section, we will describe the different regulatory mechanisms of gene expression identified so far in *Plasmodium*. Examples and specific features will be depicted in details thereafter.

### Overview of the Nucleosome Landscape

In eukaryotic cells, genomic DNA is wrapped around nucleosomes leading to the formation of a compact structure, the chromatin. A nucleosome is constituted of a histone octamer containing two units of H2A, H2B, H3, and H4. These histones can be exchanged with several histone variants that have been identified in *P. falciparum*, H2A.Z, H2B.Z, CenH3, and H3.3, and which provides an additional level of regulation (Sullivan, 2003; Miao et al., 2006; Bártfai et al., 2010). These histone variants seem to have specific features such as H2A.Z, which is associated with AT-rich sequences found in intergenic regions of *P. falciparum* (Hoeijmakers et al., 2013; Petter et al., 2013) or PfCENH3 positioned at the centromeres (Hoeijmakers et al., 2012). In addition to its packaging role, nucleosomes also play a crucial role in regulation and changes in occupancy control multiple biological processes, including gene expression. In general, active promoters of *P. falciparum* show a nucleosome-depleted region (NDR) upstream of the transcription start site (TSS), the binding site of the preinitiation complex (PIC), while silenced genes exhibit a higher nucleosome occupancy in their promoter regions hampering the interaction of the PIC (Ponts et al., 2011; Bunnik et al., 2014; Kensche et al., 2015). In model organisms, this NDR is flanked by well-positioned nucleosomes (-1 and +1) (Jiang and Pugh, 2009), but a strongly positioned nucleosome +1 is lacking in *P. falciparum* (Ponts et al., 2011; Bunnik et al., 2014). Intergenic regions display a poor nucleosome occupancy, unlike the start and end of coding regions (Westenberger et al., 2009; Ponts et al., 2011; Bunnik et al., 2014; Kensche et al., 2015), and are enriched in H2A.Z and H2B.Z (Hoeijmakers et al., 2013; Petter et al., 2013). These findings indicate that the nucleosome landscape of *Plasmodium* correspond to the initial layer in the control of gene expression.

### Overview of Epigenetic Regulation and Histone Modifiers

Although, nucleosome structure and positioning along the chromatin provide one of the first layer to control gene expression, a variety of post-translational modifications (PTMs) on the protruding N-terminal tails of histones supplement this regulatory mechanism. Histone modifications are relatively well conserved in eukaryotes including *Plasmodium*. Histone acetylation, phosphorylation and methylation are the major PTMs and can affect the interaction of the nucleosomes with DNA and the overall chromatin structure. Using such mechanism, the accessibility of promoter regions by the transcriptional machinery can be modulated, promoting or inhibiting gene expression. Several PTMs were initially detected in *P. falciparum* by mass spectrometry (Miao et al., 2006; Salcedo-Amaya et al., 2009; Trelle et al., 2009; Coetzee et al., 2017). A more recent comprehensive study identified over 230 PTMs in asexual blood stages of which 160 had never been detected in *Plasmodium* and 88 had never been identified in any other species (Saraf et al., 2016). Quantitative and dynamic profiles of histone PTMs as well as combinatorial associations indicate an unusual chromatin organization with parasite-specific histone modifications that may be directly related to transcriptional activity, DNA replication, and cell cycle progression. Overall, this data suggest that the malaria parasite has a unique histone modification signature that correlates with parasite virulence. Additional ChIP-seq experiments to identify the genome wide distribution of histone PTMs demonstrate that the histones H3K9me3 and H3K36me3 are associated with heterochromatin and silenced genes (Salcedo-Amaya et al., 2009; Trelle et al., 2009; Bunnik et al., 2018; Fraschka et al., 2018), and are mutually excluded from H3K9ac and H3K4me3, which are enriched in euchromatin and active promoters (Cui et al., 2007; Bártfai et al., 2010; Ruiz et al., 2018). These histone marks are crucial in maintaining the transcriptional state and their modification can lead to a change in the steady state. Hence, the histone PTMs can be reversed and are strictly placed under the control of a multitude of histone modifiers, the writers and erasers. The writers are enzymes capable of laying down a histone mark such as histone acetyltransferases (HATs), including GNAT and MYST proteins, and histone lysine methyltransferases (HKMTs) such as SET proteins (Cui et al., 2007; Cui et al., 2008). Their antagonists, the erasers, remove the marks placed by the writers and are composed, among others, of histone deacetylases and sirtuins (HDACs), and histone lysine demethylases (HKDMs) (Cui et al., 2008; Chaal et al., 2010). These proteins are themselves subject to PTMs, which could modulate their activity during the life cycle (Solyakov et al., 2011; Lasonder et al., 2012). Inhibition of HDACs or HATs lead to deregulation of gene expression in *P. falciparum* (Cui et al., 2007; Chaal et al., 2010), making these histone modifiers intensively studied as potential therapeutic targets in *Plasmodium* (Coetzee et al., 2020). As generally conserved across the eukaryotic phylum, additional work will be required to identify inhibitors that will target parasite enzymes with limited toxicity against their human homologs.

In addition to the histone modifiers, epigenetic marks are recognized and interpreted by proteins designated as readers, stabilizing the recruitment of specific protein complexes involved in various biological functions. Recently, several readers have been identified in *P. falciparum* using histone peptide pull-down coupled to quantitative mass-spectrometry (Hoeijmakers et al., 2019). Among them, they detected the Heterochromatin Protein 1 (HP1), well known to be associated with H3K9me3 and mediating the formation of heterochromatin (Pérez-Toledo et al., 2009). They also noticed an enrichment of bromodomain proteins PfBDP1 and PfBDP2 on acetylated H2B.Z while PfGCN5-PfADA2, members of the transcriptional coactivator complex (Cheon et al., 2020), are associated with H3K4me2/me3. Deciphering the function of writers, erasers, and readers and how they work, and form dynamic and specific complexes could be an important step in improving our understanding of gene regulation in *Plasmodium*.

Recently, the importance of noncoding RNAs (ncRNAs) as epigenetic regulators has emerged in most eukaryotes including protozoan parasites. By definition, ncRNAs are split into two groups, small (sncRNA) and long ncRNA (lncRNA) based on their respective length. In higher eukaryotes, lncRNAs influence various essential cellular processes such as chromosome maintenance, epigenetic remodeling, transcription, translation and control of protein activity (Marchese et al., 2017). In *P. falciparum*, thousands of ncRNAs have been identified but few of them have been characterized (Raabe et al., 2009; Broadbent et al., 2015). Among them, a family of telomere-associated lncRNAs (TAREs) participate in telomere maintenance and regulation of virulence gene (Broadbent et al., 2011; Sierra-Miranda et al., 2012). We describe below in more details, the role of lncRNA in the regulation of *var* genes and gametocyte commitment. Collectively, this data validates the fundamental role of epigenetic in regulation of gene expression in *Plasmodium*, through the involvement of histone marks, proteins modifiers and ncRNAs.

### Overview of Chromatin Organization

After decades of work, new evidences indicate that the chromatin architecture also plays a crucial role in eukaryotic gene expression including *Plasmodium*. Initially, fluorescence microscopy and fluorescence *in situ* hybridization (FISH) techniques have been used to investigate the chromatin organization in the parasite nucleus. Chromosome conformation capture (3C) methodology, was developed several years ago to reveal the spatial chromatin structure and nearby genomic loci (Dekker et al., 2002). The limits of this technique led to the development of derivative methods such as chromosome conformation capture-on-chip (4C) (Simonis et al., 2006; Simonis et al., 2007) and chromosome conformation capture carbon copy (5C) (Dostie et al., 2006; Ferraiuolo et al., 2012). However, to date, Hi-C is still the most used methodology to study the 3D organization of the chromosomes since it allows the identification of chromatin interactions in an “all-vs-all” manner (Lieberman-Aiden et al., 2009; van Berkum et al., 2010). Several Hi-C experiments were generated in different *Plasmodium* stages and species (Lemieux et al., 2013; Ay et al., 2014; Bunnik et al., 2018; Bunnik et al., 2019) and demonstrated distinct chromatin features at each stage of the parasite life cycle progression (see following sections for detailed characteristics). Globally, the different methods used in *P. falciparum* identified a repressive cluster at the nucleus periphery that includes telomeres and heterochromatin, while centromeres are in general found at the opposite side of the nucleus with an exception of the sporozoite stage (Freitas et al., 2005; Lopez-Rubio et al., 2009; Hoeijmakers et al., 2012; Ay et al., 2014; Bunnik et al., 2018). Although, no classical topologically associating domains (TADs) (Dixon et al., 2012; Sexton et al., 2012) were identified in *Plasmodium*, the parasite chromatin exhibits specific structure linked to virulence genes. In addition to this particular feature, a correlation between the chromatin 3D structure and gene expression profile was observed across the entire genome from the telomere to the centromere throughout the *P. falciparum* life cycle. A similar gene expression gradient was also observed in *Plasmodium knowlesi* (Bunnik et al., 2019). Conversely, no correlation between chromatin structure and gene expression profile was observed in *Babesia microti* and an inverse correlation from the centromere to the telomere was noticed in *Toxoplasma gondii*. Collectively, the spatial organization of the *Plasmodium* genomes is controlled at a higher level when compared to other Apicomplexa investigated and its dynamic throughout the parasite life cycle provides most likely a particular complementary way to control gene regulation.

### Overview of the Transcription Machinery

The three different layers of regulatory mechanisms, described above, strongly participate in the regulation of the transcription in malaria parasites, promoting or inhibiting the binding of the transcriptional machinery on the promoter and TSS regions. Indeed, in eukaryotes, the synthesis of mRNA required the recruitment of the PIC on the TSS. This complex is generally composed of the TATA-binding proteins (TBPs), TBP-associated factors (TAFs), the general transcription factors TFII, and the RNA polymerase II (RNA pol II) (Luse, 2014). In *Plasmodium*, despite the presence of most of the components of this complex, some subunits have not yet been identified suggesting a partial adaptation of the parasite to ensure a proper transcription (Callebaut et al., 2005). Moreover, some of the components of the transcriptional machinery such as PfTBP and PfTFIIE have been detected upstream of inactive genes lacking acetylated histones (Gopalakrishnan et al., 2009). Similarly, RNA pol II has been detected in early and late asexual stages on active and inactive promoters (Rai et al., 2014). It has been suggested that pausing mechanism of the RNA pol II at these stages could be a major way to control transcriptional activation and elongation (Lu et al., 2017). These initial results indicate that PIC occupancy is not clearly associated with the transcriptional status and multiple mechanisms may be involved to activate the *Plasmodium* transcriptional machinery.

In addition to the PIC, specific transcription factors are key proteins promoting or inhibiting gene’s transcription. Through their DNA-binding domains, they are able to bind specific DNA motifs, identified as enhancers or promoter regions to recruit chromatin modifying and remodeling complexes as well as the PIC when needed. The ratio of the total number of genes to the predicted number of specific transcription factors in *P. falciparum*, is one of highest in eukaryotes indicating a relative paucity of transcription factors (Templeton et al., 2004). The discovery of the 27 members of the ApiAP2 transcription factor family or AP2s, specific to apicomplexan parasites and analogous to Apetala-2 in plant, was instrumental to our understanding of gene regulation in *Plasmodium* (Balaji et al., 2005; Campbell et al., 2010). Due to their specificity, they have been the subject of particular attention to comprehend how so few transcription factors could tightly control the expression of 5,500 coding genes during the entire life cycle. One hypothesis is that these AP2s operate as master regulators and transcribe hundreds of genes at specific stages. This has been specifically demonstrated for the transcription factors involved in stage transition such as AP2-G and AP2-G2 in gametocytes (Sinha et al., 2014; Yuda et al., 2015), AP2-O in ookinetes (Yuda et al., 2009), AP2-SP in sporozoites (Yuda et al., 2010) and AP2-L in liver stages (Iwanaga et al., 2012). Recently, knock-out screenings in rodent *Plasmodium* have showed that these AP2s are essential to these particular stages during parasite development (Modrzynska et al., 2017; Zhang et al., 2017). However, a recent machine learning model demonstrated that at least in asexual stages, the identified AP2 DNA-binding motifs, may play a limited role in erythrocytic transcriptional regulation suggesting that while AP2 may interact with some promoters to either act as a repressor or activator such interaction can only happen in association with several chromatin-associated proteins and a favorable epigenetic environment to ensure transcription (Read et al., 2019). Taken together, *Plasmodium* parasites have developed some particular features to compensate for their lack of transcription factors.

## Gene Regulation During Intraerythrocytic Developmental Cycle

### Transcriptional Status Across the Intraerythrocytic Developmental Cycle

The IDC of *P. falciparum* has been well studied over the years since it is responsible for most symptoms observed in humans. Typically, this cycle is defined by rings, trophozoites, and schizonts but correlating these phenotypic stages to classical eukaryotic cell cycle phases (G1, S, G2, and M) has been quite challenging (Arnot et al., 2011).

**Figure 1:**
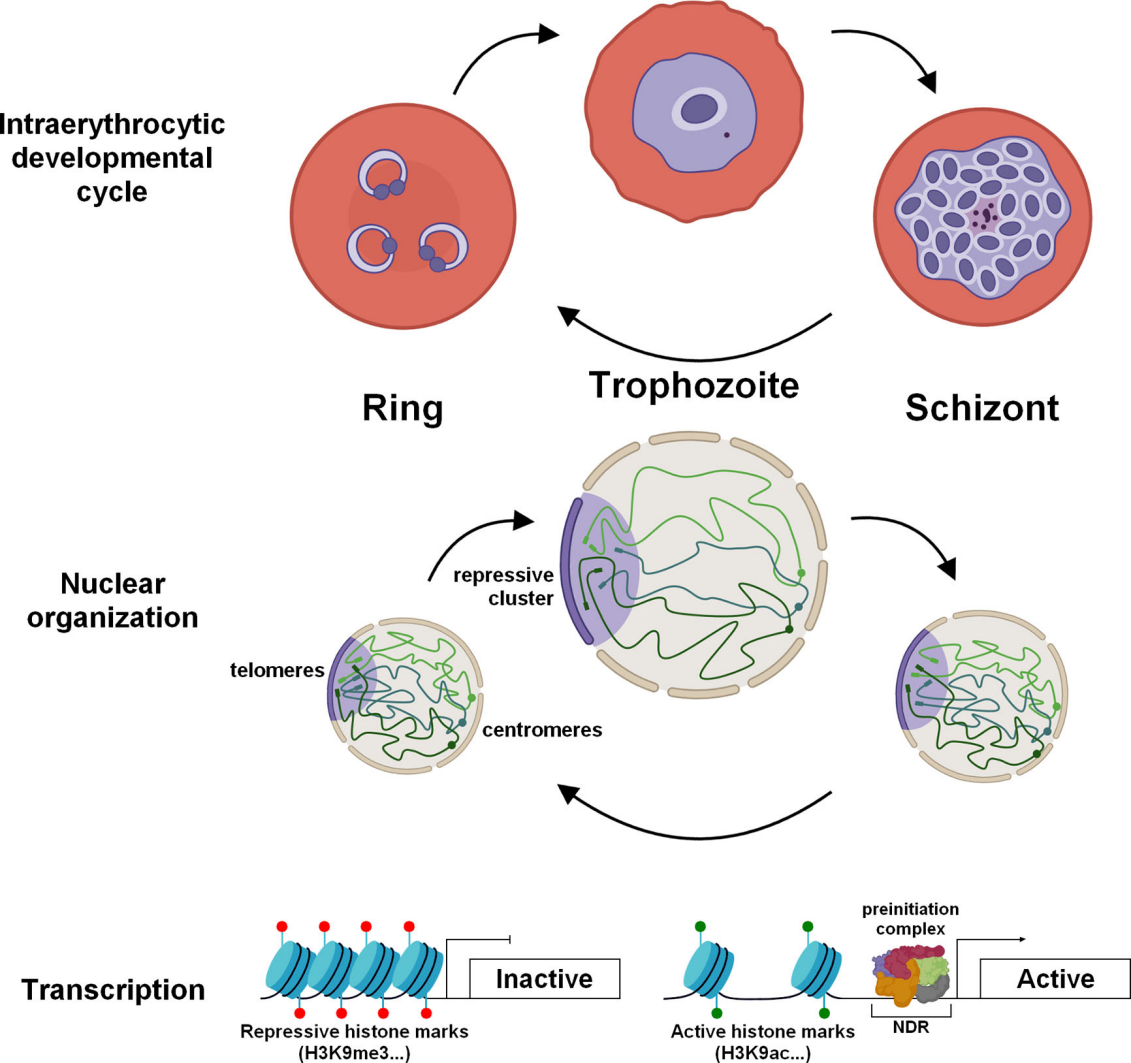
Dynamic chromatin architecture and epigenetic regulation in asexual blood stages of *P. falciparum*. During the IDC, drastic re-organization of the chromatin is observed to promote the transcriptional burst in trophozoite stage. The chromatin is lightly packed and enriched in active histone marks. The size of the nucleus increases as well as the number of nuclear pores. Throughout the IDC, centromeres (spheres) are clustered and located at the periphery of the nucleus while telomere regions (rectangles) form a repressive cluster on the opposite side.

In ring stage, the nucleus is globally compacted (Weiner et al., 2011; Ay et al., 2014) and chromosomes are enriched in nucleosomes (Ponts et al., 2010; Bunnik et al., 2014) (**Figure 1**). This condensed environment may inhibits transcription and result in low transcriptional activity observed at this stage (Bozdech et al., 2003; Le Roch et al., 2003). To complement nuclear compaction, the number of nuclear pores detected is low and their size relatively small, which correlate with low transcriptional activity and RNA import/export (Weiner et al., 2011). Moreover, pausing of RNA pol II has been detected at this specific stage suggesting that while the polymerase is positioned on promoter regions in anticipation of the trophozoite stage, it is not activated for transcriptional initiation and elongation processes (Lu et al., 2017).

The trophozoite is considered as the principal stage of development with significant morphological changes and remodeling of the infected red blood cell. During this step, the volume of the genome increases considerably indicating a more open chromatin structure (Ay et al., 2014), in correlation with an active transcription status (Bozdech et al., 2003; Le Roch et al., 2003) (**Figure 1**). This is also accompanied by a notable increase in the number of nuclear pores, which exhibit a specific distribution and are enriched in transcriptionally active compartments to facilitate RNA export (Weiner et al., 2011; Dahan-Pasternak et al., 2013). To ensure an intense transcriptional activity (Lu et al., 2017), this stage undergoes drastic chromatin rearrangement such as decrease of nucleosome occupancy in promoter regions (Ponts et al., 2010; Bunnik et al., 2014; Kensche et al., 2015) and modifications of histone marks with presence of H3K9ac and H3K4me3 in promoter of active genes (Cui et al., 2007; Bártfai et al., 2010; Ruiz et al., 2018) (**Figure 1**). Conversely, genes known to be critical in sexual and mosquito stages exhibit a high nucleosome occupancy and repressive histone PTMs.

During the schizogony, nucleosomes are repacked promoting the compaction of the chromatin, required for the formation of daughter cells and the next invasion (Ponts et al., 2010; Ay et al., 2014; Bunnik et al., 2014). Global transcription is also highly reduced and a decrease in the size and number of nuclear pores is observed (Weiner et al., 2011) (**Figure 1**). Despite intensive nuclear compaction, genes involved in invasion, such as erythrocyte binding antigens, merozoite surface proteins, and rhoptry associated proteins, are highly transcribed at this stage (Le Roch et al., 2003; Lu et al., 2017). This transcription is controlled by PfBDP1, in association with the transcription factor AP2-I, and the acetylated histones enriched in their promoter regions (Josling et al., 2015; Santos et al., 2017). Analysis of the schizont transcriptome from field and laboratory strains showed a differential expression of these invasion genes, suggesting the adaptation of parasites to successfully invade red blood cells (Tarr et al., 2018). Machine learning model suggested that high levels of H3K9ac and H4K20me3 marks correlate strongly with high expression in schizonts, while H3K4me3 marks correlate to active genes in ring and trophozoite stages (Read et al., 2019). After a schizont burst, the merozoites are released into the bloodstream and invade new red blood cells, which perpetuates the erythrocytic cycle. Although few studies are available on gene regulation at this stage, we can assume that the genome is highly compacted, as the function and size of these parasites seem to indicate. The lowest level of transcription in the entire life cycle was also detected at the population and single-cell levels and also corresponds to invasion genes (Bozdech et al., 2003; Le Roch et al., 2003; Howick et al., 2019). Altogether, the data validate a strong and tight gene regulation across the IDC consistent with the role of these four asexual stages.

Transcriptome analyzes of resistant strains showed that parasites can adjust their gene expression profile and life cycle in response to drugs by slowing or arresting their cell cycle to protect their survival (Adjalley et al., 2015; Shaw et al., 2015; Rocamora et al., 2018). Recent studies using RNA-seq on field isolates causing more severe malaria showed a distinct transcriptome with upregulation of genes involved in multiples pathways such as pyrimidine metabolism, tricarboxylic acid cycle and GTPase activity while *var* genes were down-regulated (Tonkin-Hill et al., 2018). Collectively, these studies indicate that environment changes can have a significant effect on *P. falciparum* gene expression across its life cycle.

### Regulation of Virulence Genes

**Figure 2:**
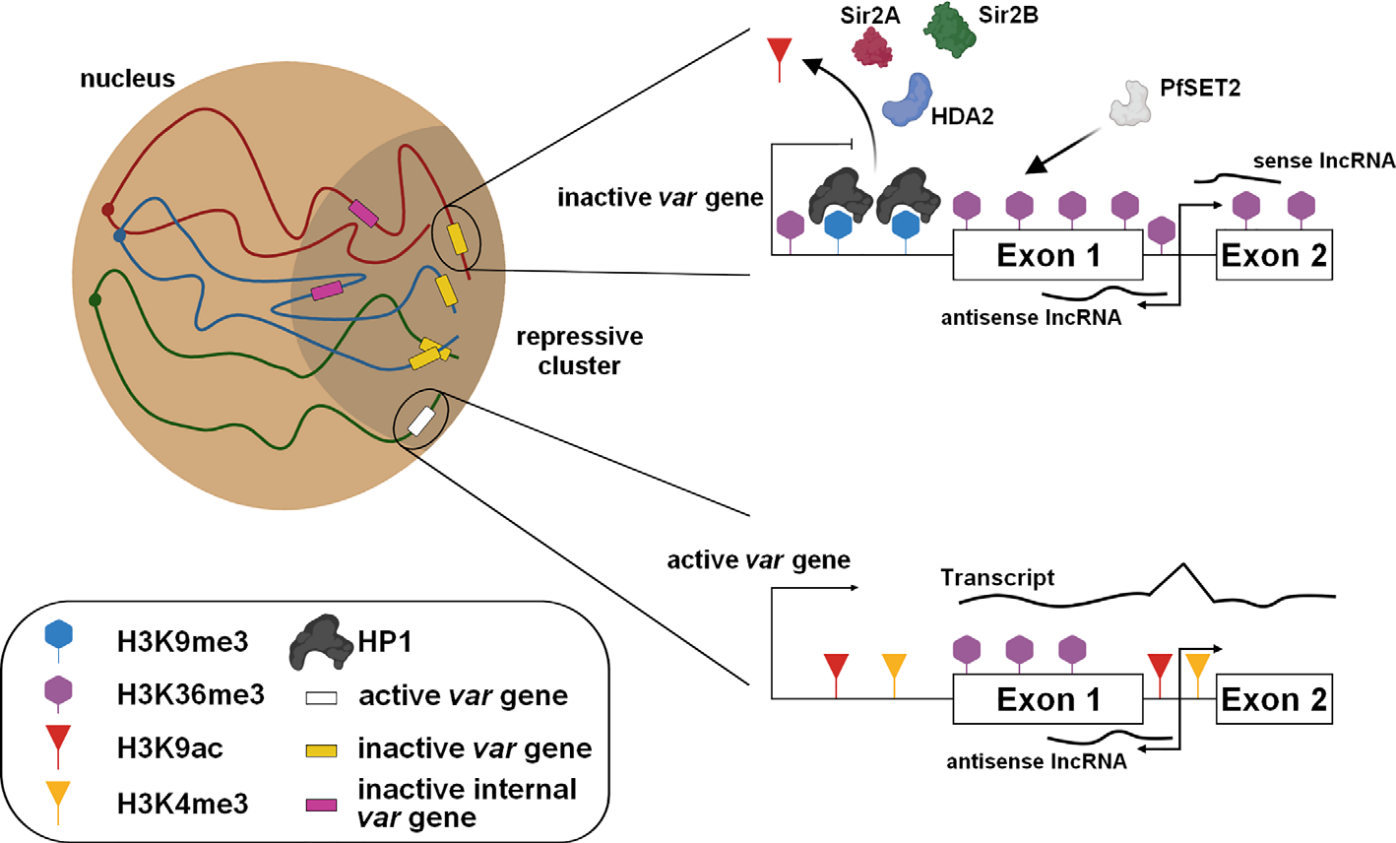
Regulation of the mutually exclusive *var* gene expression. The subtelomeric and internal *var* genes are grouped in the repressive cluster and maintained in a silent state with the presence of repressive marks and the expression of specific lncRNAs. For the active *var* gene present at the nuclear periphery, epigenetic changes occur and require the involvement of protein modifiers.

One of the most interesting features of the parasite is its ability to evade the host’s immune system. For this purpose, *P. falciparum* possesses several clonally variant gene families such as *var*, *rifin*, *stevor*, and *Pfmc-2TM*, and most of these genes are located in the subtelomeric regions. The most studied family is that of *var*, which contains ~ 60 genes encoding PfEMP1 antigen, mediating cytoadherence and infected red blood cells sequestration. Only one copy of PfEMP1 is expressed at a time and exported at the surface of the infected red blood cell, limiting the exposure of PfEMP1 variants to the host immune system. Thus, this mutually exclusive expression requires a tight regulation to ensure that all *var* genes are repressed, while only one *var* gene is actively transcribed and translated. In asexual blood stages, inactive *var* genes are enriched in H2A as well as the repressive marks H3K9me3 and HP1 (Freitas et al., 2005; Chookajorn et al., 2007; Flueck et al., 2009; Lopez-Rubio et al., 2009; Salcedo-Amaya et al., 2009; Trelle et al., 2009; Ukaegbu et al., 2014). These genes are present at the periphery of the nucleus, and regrouped at the repressive cluster(s) (Ralph et al., 2005; Lopez-Rubio et al., 2009; Ay et al., 2014; Bunnik et al., 2019) (**Figure 2**). Conversely, the active *var* gene is also present at a perinuclear location, permissive for transcription, and showed a high distribution of H3K9ac and H3K4me3 marks, and H2A.Z/H2B.Z histone variants (Freitas et al., 2005; Lopez-Rubio et al., 2007; Petter et al., 2011; Petter et al., 2013). Moreover, histone PfH3.3 is associated with the active promoter and could play a role in memory for the next generation of parasites (Fraschka et al., 2016). While most of the *var* genes are localized in telomeric regions of all chromosomes, some can be found in the core of the chromosomes. For those, the formation of large chromatin loops facilitate their clustering and interaction with all subtelomeric *var* loci (Lemieux et al., 2013; Ay et al., 2014).

Several histone modifiers have been described to be directly involved in modifications of these histone marks localized on the *var* gene loci (**Figure 2**). The histone deacetylases PfSir2A, PfSir2B, and HDA2 and the methyltransferases, PfSET10 and PfSET2, have been identified to be essential in the poise of *var* expression (Duraisingh et al., 2005; Freitas et al., 2005; Lopez-Rubio et al., 2009; Volz et al., 2012; Jiang et al., 2013; Coleman et al., 2014; Ukaegbu et al., 2014). An alternative regulatory mechanism was also identified with the involvement of sense and antisense lncRNAs transcribed from the intron and extending to exons 1 and 2 (Epp et al., 2009) (**Figure 2**). The antisense lncRNA is associated with its locus to activate the gene while its interference causes a decrease of active *var* gene expression, promoting a switching (Amit-Avraham et al., 2015; Jing et al., 2018). Furthermore, a family of GC-rich ncRNA is known to act in *trans-* and *cis-* to regulate the repressed *var* genes as well as the active locus (Wei et al., 2015; Guizetti et al., 2016).

The family of clonally variant gene *Pfmc-2TM* is also placed under the control of these GC-rich regulatory elements (Barcons-Simon et al., 2020) while the transcription factor AP2-exp has been recently implicated in the expression of *rifin* and *stevor* (Martins et al., 2017). Altogether, the data showed that the mutual exclusive expression of virulence genes, required to escape the immune response, is tightly regulated by epigenetic factors and chromatin architecture.

Mutual exclusive expression is also described in invasion genes such as clag3.1 and clag3.2, for which histone marks and noncoding RNAs are involved (Cortés et al., 2007; Rovira-Graells et al., 2015) and this epigenetic memory seems to be erased during the conversion into transmission stages (Mira-Martínez et al., 2017).

## Commitment to Sexual Stages

**Figure 3:**
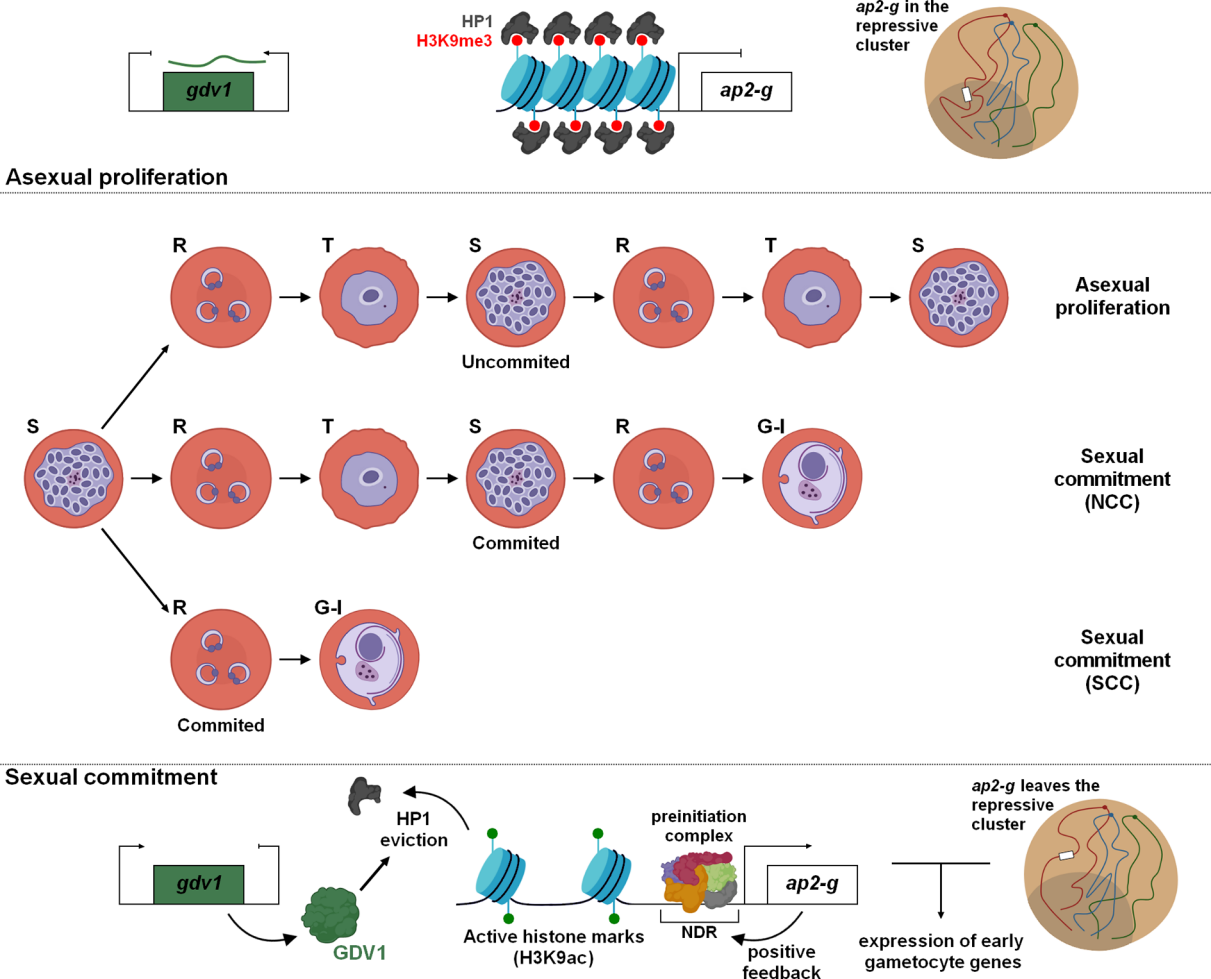
Sexual commitment in *Plasmodium falciparum*. Cellular and molecular mechanisms maintain in a poised state the sexual and asexual proliferation. Epigenetic regulation and global changes of chromatin structure are fundamental for the expression of PfAP2-G and initiation of gametocytogenesis. Depending on the timing of PfAP2-G stabilization, two differentiation pathways have been identified: NCC, Next Cycle Conversion and SCC, Same Cycle Conversion.

During the IDC, a proportion of parasites differentiate into sexually mature male and female gametocytes. As these forms are essential for transmission of the parasite into mosquitoes, interest has grown over the years to better understand the molecular mechanisms driving the conversion and the development of gametocytes with the hope that researchers will identify new drugs or vaccine strategies to block disease transmission. A few years ago, AP2-G was identified and validated as a master transcription factor for sexual commitment in *Plasmodium* spp. (Kafsack et al., 2014; Sinha et al., 2014; Kent et al., 2018). In the asexual cycle, *ap2-g* is repressed and its promoter enriched in repressive histone marks (e.g H3K9me3) as well as HP1 (Brancucci et al., 2014; Coleman et al., 2014; Filarsky et al., 2018) (**Figure 3**). Various environmental factors such as food, lysophosphatidylcholine restriction, and high parasitemia have been described to be preponderant in the sexual development (Brancucci et al., 2017), but others factors, signaling pathways, and metabolites remain most likely to be discovered. Despite uncertainties, several studies have demonstrated the crucial role of Gametocyte development protein 1 (GDV1) during sexual differentiation. In asexual blood stages, the expression of GDV1 is repressed by its own antisense RNA, while in stress conditions, an unknown mechanism restrains the function of the inhibitory RNA leading to GDV1 expression (**Figure 3**). This protein triggers HP1 eviction on the *ap2-g* promoter, which destabilizes silencing leading to de-repression and activation of *ap2-g* expression (Broadbent et al., 2015; Filarsky et al., 2018). After a first peak, a rapid drop of AP2-G transcripts abundance is observed before a new wave of expression (Poran et al., 2017). A positive transcriptional feedback loop of AP2-G participates in this stabilization and ensure a bistability switch (Poran et al., 2017), a model of all-or-none expression described in several cell differentiations (Xiong and Ferrell, 2003; Wang et al., 2009; Bhattacharya et al., 2010). The ApiAP2 transcription factor, PF3D7_1222400, adjacent to *ap2-g* locus, appears to participate in this switch as well as the helicases ISWI and SNF2L, whose the expression is correlated with the de-repression of *ap2-g*, and could contribute to the accessibility of the locus (Poran et al., 2017). If this stabilization occurs in early ring stage, the parasite can directly start its differentiation in gametocyte, designated as Same Cycle Conversion (SCC) route (Bancells et al., 2019). Otherwise, the Next Cycle Conversion (NCC) is observed and parasite pursues its development until schizogony and invasion before to initiate the sexual development. Regarding *ap2-g* locus, Hi-C result indicates that the gene is no longer associated with the repressive territory in early gametocytes (Bunnik et al., 2018). We can assume *ap2-g* leaves this region upstream the sexual commitment to facilitate its expression (**Figure 3**).

The binding of AP2-G to specific motifs of hundreds of active promoters designates it as the master regulator of gametogenesis (Campbell et al., 2010; Kafsack et al., 2014; Sinha et al., 2014; Poran et al., 2017; Kent et al., 2018; Josling et al., 2020). These target genes are mostly considered as early gametocyte genes or secreted proteins involved in erythrocyte remodeling (Kafsack et al., 2014; Poran et al., 2017). In committed schizonts, AP2-G was also detected upstream of some invasion genes such as *sera*, *eba175*, and *ron5*, occasionally in cooperation with AP2-I (Poran et al., 2017; Josling et al., 2020). Further investigations could provide valuable information to decipher this conversion mechanism and facilitate the identification of potential therapeutic targets to hamper the parasite transmission.

## Gene Regulation During Gametocytogenesis

Once parasites are committed, immature forms develop into male and female gametocytes during the gametocytogenesis. The duration of this process differs between the different species of *Plasmodium*. The maturation of gametocytes takes 24–48h in the rodent parasites, while 8-12 days are necessary for *P. falciparum*, divided in 5 stages (I to V), morphologically distinguishable (Gautret and Motard, 1999).

Although, the telomeres and centromeres are still grouped in their respective cluster, a reorganization of the chromosomes is detected by Hi-C in gametocytes (Bunnik et al., 2018). The heterochromatin cluster is expanded and contains virulence and invasion genes as well as the genes not required for the gametocytogenesis and an enrichment of the repressive marks H4K20me3, H3K27me3 and H3K36me2 is observed in stage I-III gametocytes (Coetzee et al., 2017). Interestingly, some histone modifications are described to be specific to gametocytes such as H3K36me2/me3, H3K27me2/me3, and H3K79me3 while H3K9me2, H3K18me1, and H3K4me2/me3 are specific of asexual stages (Coetzee et al., 2017).

In late gametocytes, interactions between *ap2-g* locus and virulence genes were detected by Hi-C, suggesting the gene has regained its place in the repressive cluster, at least partially (Bunnik et al., 2018). Another transcription factor, AP2-G2, has a specific function in gametocytes by repressing genes required for asexual proliferation since mutant parasites in *P. berghei* are able to differentiate in gametocytes but cannot fully mature (Yuda et al., 2015).

Hi-C experiment identified additional large chromosome rearrangements on chromosome 14, with the formation of two super domains (Bunnik et al., 2018), similar to what is observed during the inactivation of one of the X chromosome in human and mouse (Rao et al., 2014; Deng et al., 2015). Near the boundary of these two super domains, *ap2-o3* and *ptpa* have been identified and both are involved in sexual development suggesting this large rearrangement may promote their active transcription. The transcription factor AP2-O3 is described as specific of female gametocytes and is required for normal ookinete formation in rodent parasites (Modrzynska et al., 2017; Zhang et al., 2017) while PTPA regulates the activity of the phosphatase PP2A and participates in the regulation of the *P. falciparum* cell cycle (Vandomme et al., 2014).

During the maturation process, parasites differentiate sexually in phenotypically distinguishable male and female gametocytes. It is still unclear whether the sexual determination occurs with the early sexual commitment or if it is happening downstream (Tadesse et al., 2019). Interestingly, the sex ratio seems to fluctuate according to environmental conditions and *Plasmodium* species, but overall, the balance is in favor of females (Tadesse et al., 2019). Thereafter, male gametocytes will be activated and form eight microgametes in the mosquito midgut, counterbalancing the previous imbalance (Kuehn and Pradel, 2010).

Despite differences in their respective transcriptomes and proteomes (Khan et al., 2005; Walzer et al., 2018), few epigenetic changes were observed between female and male gametocytes in *P. berghei* (Witmer et al., 2020). The histone mark H3K9ac is associated with active transcription in asexual blood stages and male gametocytes, while female gametocytes do not show a higher occupancy and a low abundancy in histone variants H2A.Z and H2B.Z is also detected (Witmer et al., 2020). This result could be explained by the storage of the mRNAs in a messenger ribonucleoprotein complex and the involvement of mRNA binding proteins to regulate transcription at the post-transcriptional level at this particular stage. Additionally, a transcription factor, identified as AP2‐G3 or AP2‐FG, has been described as female specific and demonstrated to regulate over 700 genes (Yuda et al., 2019). Collectively, different regulatory changes were observed during the gametocytogenesis and participate in both sexual development and differentiation between female and male gametocytes.

## Gene Regulation in Mosquito and Liver Stages

The immature gametocytes, stages I to IV of *P. falciparum*, are mainly sequestrated in bone marrow and cannot be detected in peripheral circulation in infected humans (Gardiner and Trenholme, 2015). Mature stage V gametocytes re-enter into the bloodstream and can be ingest by a mosquito during a blood meal. Once gametocytes reach the mosquito’s midgut, environmental signals activate the gametogenesis (Kuehn and Pradel, 2010) and formation of zygote, ookinete, and then oocyst. Due to the difficulty to isolate these stages, few studies are available. Epigenetic analyzes in oocysts of *P. falciparum* and *P. berghei* showed the heterochromatin distribution is globally conserved and H3K9ac and H3K27ac are highly correlated with active transcription (Gómez-Díaz et al., 2017; Witmer et al., 2020). An interesting example of genetic regulation concerns the protein Cap380, a marker of oocyst development, whose gene is not expressed during IDC and gametocytes (Itsara et al., 2018). In oocysts, an enrichment in H3K9ac was observed in the promoter region of *pbcap380*, in correlation with a higher abundance of *cap380* transcripts (Witmer et al., 2020).

Several ApiAP2 transcription factors have been demonstrated as essential for the parasite development such as AP2-O and AP2-O2 in *P. falciparum* and rodent parasites (Yuda et al., 2009; Kaneko et al., 2015; Modrzynska et al., 2017; Zhang et al., 2017). In *P. berghei*, AP2-O was detected by ChIP-seq in promoter regions of more than 500 genes, including *cap380*, confirming the role of this protein as master regulator (Kaneko et al., 2015).

In *P. falciparum* and *P. vivax* sporozoites, Hi-C experiments indicated that invasion and virulence genes are strongly associated with the repressive cluster (Bunnik et al., 2018). These genes showed an enrichment of H3K9me3 as well as telomeric and subtelomeric regions while H3K9ac and H3K4me3 are present along the chromosomes, especially outside the coding regions for *P. vivax* (Gómez-Díaz et al., 2017; Zanghì et al., 2018; Vivax Sporozoite Consortium, 2019). Interestingly, one *var* gene (PF3D7_1255200) seems to play a specific role at this stage. Compared to other virulence genes, *pf3d7_1255200* showed low level of H3K9me3 and an AP2-exp binding motif, known to control expression of a subset of clonally variant gene families (Gómez-Díaz et al., 2017; Martins et al., 2017). In addition, the expression of the lncRNA antisense correlates with the expression of this gene confirming the involvement of lncRNA in the *var* regulation (Gómez-Díaz et al., 2017).

Hi-C experiment in P. falciparum sporozoites demonstrated a chromatin rearrangement with loops and long-range interactions for csp, trap, and spect1 (Bunnik et al., 2018). Likewise, PbAP2-SP was detected in promoter regions of sporozoite‐specific genes such as spect1, trap, and sera, and its disruption prevented sporozoite formation confirming the essentiality of this factor (Yuda et al., 2010). Additional transcription factors were identified to play a crucial role in sporozoite development such as AP2-SP2 and AP2-SP3 (Modrzynska et al., 2017; Zhang et al., 2017). Rearrangements are also observed with the loss of the centromeres cluster and the A-type rDNAs showed a large increase in contacts with the repressed virulence genes, transcriptionally inactive in mosquito stages (Bunnik et al., 2018). Altogether, to ensure the transcription of genes required for cell traversal and hepatocyte invasion, chromatin is significantly reorganized to facilitate the involvement of specific transcription factors.

Due to the difficulty of working at the liver stage, regulation of gene expression has been very little studied. In *P. cynomolgi*, responsible of malaria in various macaque monkeys, the use of a histone methyltransferase inhibitor in liver culture was able to promote hypnozoite activation, suggesting that epigenetic marks could be involved in parasite reactivation, although its direct effect has not yet been demonstrated (Dembélé et al., 2014). One AP2 transcription factor, PbAP2-L, was demonstrated as not essential for liver invasion but crucial for the expression of several genes and maturation of the parasite inside hepatocytes (Iwanaga et al., 2012). Further experiments are needed to decipher the exact mechanisms regulating gene expression in liver stage and highlight possible specific features.

## Discussion

In this review, we covered the recent progress on regulation of gene expression in *P. falciparum*. This regulation encompasses local and global chromatin structure changes across the whole life cycle to ensure the proper transcription and stage conversion. Local modifications play a primordial role with the involvement of nucleosome occupancy, epigenetic modifications, protein modifiers, and lncRNAs promoting gene activation or silencing. Heterochromatin is enriched in HP1, H3K9me3, and H3K36me3 marks while euchromatin exhibits H3K9ac and H3K4me3 marks and a lower nucleosome occupancy facilitating the binding of the general transcriptional machinery in the NDR. During the IDC, drastic chromatin remodeling has been observed in relation to the transcriptional status of each stage. Unlike compact chromatin at the ring and schizont stages, an open chromatin was observed in trophozoites by Hi-C and FISH experiments promoting the transcriptional burst. The exploration of the regulation of virulence genes and in particular the mutually exclusive expression of *var* genes highlighted how genes can be controlled by a combination of different and complementary mechanisms. Although the overall architecture is similar in gametocytes, few exceptions were detected as the presence of specific histone marks and the remodeling of the *ap2-g* locus. This master transcription factor is essential for the sexual commitment and epigenetic modifications are required on its locus and that of *gvd1*, as well as a re-organization from the repressive cluster. In mosquito and liver stages, few studies have been conducted but particular features have been observed such as the loss of the centromere clustering in sporozoites. However, our understanding of gene regulation in *Plasmodium* is far from complete and further experiments are still require to decrypt all molecular components and specific features involved in controlling parasite development, in particular in stages that are under investigated such as in mosquito or liver.

## Author Contributions

TH and KLR conceived and wrote the manuscript. All authors contributed to the article and approved the submitted version.

## Funding

This work was supported by the National Institutes of Allergy and Infectious Diseases of the National Institutes of Health (grant R01 AI136511 and R21 AI142506-01 to KLR) and the University of California, Riverside (NIFA-Hatch-225935 to KLR).

## Conflict of Interest

The authors declare that the research was conducted in the absence of any commercial or financial relationships that could be construed as a potential conflict of interest.
